# Challenges regarding informed consent in recruitment to clinical research: a qualitative study of clinical research nurses’ experiences

**DOI:** 10.1186/s13063-023-07844-6

**Published:** 2023-12-11

**Authors:** Tove Godskesen, Joar Björk, Niklas Juth

**Affiliations:** 1 Centre for Research Ethics & Bioethics, Department of Public Health and Caring Sciences, Uppsala University, Uppsala, Sweden; 2 Faculty of Nursing and Health Sciences, Nord University, Bodø, Norway; 3 Stockholm Centre for Healthcare Ethics (CHE), LIME, Karolinska Institutet, Stockholm, Sweden; 4 Department of Research and Development, Region Kronoberg, Växjö, Sweden

**Keywords:** Clinical research, Informed consent, Nursing ethics, Research ethics, Qualitative content analysis

## Abstract

**Background:**

Clinical research nurses (CRNs) have first-hand experience with ethical challenges and play a crucial role in upholding ethical conduct and adherence to the principles of informed consent in clinical research. This study explores the ethical challenges encountered by CRNs in the process of obtaining informed consent for clinical research.

**Methods:**

A qualitative exploratory design. Semistructured interviews (*n* = 14) were conducted with diverse CRNs in Sweden. These CRNs covered a wide range of research fields, including pharmaceutical and academic studies, interventions, and observational research, spanning different trial phases, patient categories, and medical conditions. The interviews were analysed using inductive qualitative content analysis.

**Results:**

The analysis identified three main categories: (i) threats to voluntariness, (ii) measures to safeguard voluntariness, and (iii) questionable exclusion of certain groups. CRNs face challenges due to time constraints, rushed decisions, information overload, and excessive reliance on physicians’ recommendations. Overestimating therapeutic benefits in stages of advanced illness emerged as a risk to voluntariness. CRNs outlined proactive solutions, such as allowing ample decision-making time and offering support, especially for terminally ill patients. Concerns were also voiced about excluding certain demographics, such as those with language barriers or cognitive impairments.

**Conclusions:**

In conclusion, upholding ethical research standards requires recognising various factors affecting patient voluntariness. Researchers and CRNs should prioritise refining the informed consent process, overcoming participation challenges, and aligning scientific rigour with personalised care. Additionally, a concerted effort is vital to meet the diverse needs of patient populations, including equitable inclusion of individuals with language barriers or cognitive limitations in clinical studies. These findings have significant implications for enhancing the ethics of clinical research and advancing person-centred care.

**Supplementary Information:**

The online version contains supplementary material available at 10.1186/s13063-023-07844-6.

## Background

Informed consent is a crucial ethical and legal obligation in human subject research. Striving for informed consent serves two primary objectives: (i) respecting and supporting the participant’s autonomy and (ii) ensuring protection from potential harm. Obtaining written informed consent from participants before their enrolment in a study is an internationally recognised standard practice widely acknowledged and endorsed [1–4]. The role of registered nurses (RNs) working as clinical research nurses (CRNs) is often claimed to be invaluable to the research process [5]. As essential research team members, CRNs should uphold ethical principles throughout the research process, recognising that informed consent is more than a mere formality — a fundamental requirement deeply rooted in ethics and law.

To ensure the ethical validity of informed consent, three fundamental requirements must be met [6]. First, potential research subjects must comprehend the provided information to make an informed decision about their participation [7]. CRNs, along with other research team members, bear the responsibility of providing complete and comprehensive information regarding the research purpose, procedures, potential risks or discomforts, and the participant’s right to withdraw from the study at any time.

Second, research participants must be able to make their own free decision to participate without any undue influence, pressure or coercion affecting them [7, 8]. CRNs play a crucial role in delivering clinical research [9, 10] and have an active part in the informed consent process [5].

The third requirement is that participants must possess the capacity to provide voluntary and explicit consent for their participation. CRNs play a crucial role in assessing participants’ understanding and ensuring that they comprehend the information presented to them. They must consider various factors that may hinder participants’ comprehension, such as personal fears, the impact of their disease, anxiety, pain, and suffering [11]. This becomes particularly important when working with vulnerable populations, such as children or individuals with disabilities; CRNs are responsible for ensuring the protection of participants’ rights and welfare [12]. Still, it is the PI/CI who bears the ultimate responsibility for overseeing and ensuring the ethical conduct of the study. Their collaboration with CRNs is pivotal in achieving this goal.

The informed consent process is not a one-time event; it marks the beginning of a relationship built on effective communication, and CRNs should strive to foster this relationship [13]. The informed consent process is not a one-time event; it marks the beginning of a relationship built on effective communication, and CRNs should strive to foster this relationship. Importantly, informed consent is an ongoing process that spans the entirety of a clinical research study, encompassing both the initial consent procedure and all subsequent interactions related to consent with participants. Continuous consent entails sustained communication with research participants, ensuring that they remain well-informed about the study’s progress, any revisions in procedures, and potential risks or benefits. It also includes additional consent if new information arise that might influence a participant’s decision to continue their involvement in the trial [14]. CRNs should strive to maintain participants’ trust, protect their confidentiality, and ensure their rights and well-being throughout the study. However, CRNs also face ethical challenges during the informed consent process. Unrealistic expectations about the study’s benefits or concerns regarding the vulnerability of end-of-life patients can create dilemmas for CRNs [15–17]. Balancing the core values of nursing care, such as prioritising patient well-being and autonomy, which are grounded in established nursing ethics codes and professional guidelines, and recruiting an adequate number of participants can present significant challenges for CRNs. These values, as outlined by governing body codes of conduct and nursing standards, encompass principles that emphasise patient-centred care, respect for autonomy, and ensuring the highest standards of patient well-being. Striking the right balance between these priorities requires careful navigation and decision-making to ensure ethical conduct and meaningful research outcomes [13, 18].

The International Council of Nurses [19] emphasises the significance of authorising and preparing RNs for research. Despite the crucial role played by CRNs in the informed consent process, there is a notable lack of research investigating their experiences, perspectives, and challenges while fulfilling this essential role [20]. Thus, this study explores ethical challenges encountered by CRNs in the process of obtaining informed consent for clinical research.

## Methods

### Design

This study utilised a qualitative exploratory design [21]. The current study adheres to the consolidated criteria for reporting qualitative research (COREQ) checklist as outlined by Tong et al. [22] (Additional file 1).

### Setting

In Sweden, as in many other countries, the primary responsibility for all trial-related medical decisions lies with the PI/CI, who is often a specialist in a specific field like oncology, and this practice aligns with Good Clinical Practice guidelines [23]. Within the research team, the principal investigator and sub-investigators oversee the trial, ensuring its integrity, while the regulatory coordinator ensures adherence to regulations. Typically, research teams in most projects consist of doctors, research nurses, project coordinators, and secretaries. Among these roles, CRNs play a pivotal role. They actively engage with patients, explaining the details of participation, closely monitoring trial participants, collecting clinical data, and promptly notifying the investigator of any adverse events or health concerns. CRNs often serve as the primary point of contact for participants and are instrumental in facilitating effective communication between participants and the investigator regarding their medical care needs [5, 18].

This study categorises research studies as interventional or observational. Interventional studies evaluate the effectiveness of interventions such as drugs or procedures, while observational studies analyse existing behaviours or relationships. Interventional studies typically have four phases: Phase I involves initial testing of a drug or treatment in a small group to assess safety and dosage. Phase II evaluates effectiveness and safety in a larger group, and Phase III confirms efficacy, monitors side effects, compares to existing treatments, and ensures safe usage. A phase IV study, also known as post-marketing surveillance, is conducted after a treatment has been approved by regulatory authorities to monitor its safety and effectiveness in a larger population and over an extended period.

### Recruitment and participants

The study used maximum variation sampling and purposive snowball sampling to recruit participants who worked as CRNs in Sweden. The first author obtained two lists of network members, including names and email addresses, from the head of the CRN networks, as there is no Swedish register specifically for CRNs [5]. Participants were purposively invited to take part in the study via email. The aim was to achieve a diverse sample representing different types of research studies and varied years of experience in research. Out of the initial 62 CRNs on the list, a subset of nurses was invited to participate in the study based on criteria that included geographic diversity and affiliations with small or large university hospitals. By employing this sampling method, the study could capture perspectives from diverse clinical units, spanning both pharmaceutical and academic studies, interventions and observational research, various trial phases, and a wide range of patient populations and diseases (Table 1). Participants were sent inclusion criteria and study details, which covered confidentiality and voluntary participation, via email upon expressing interest. If they met the criteria and were interested, an interview appointment was scheduled via email. All participants provided written informed consent either in person before the interview or electronically via email.

**Table 1:** Characteristics of clinical studies in which CRNs (*n* = 14) were involved

**Examples of study focus**	**Sponsor**		**Study design**	
Depression Diabetes Gastroenterology Gynaecology Heart Kidney Lung Neurology Obesity Oncology Parkinson Surgery Urology	Pharmaceutical industry research	12	*Intervention studies:*	
	Academic research	13	Phase I	5
			Phase II	7
			Phase III	10
			Phase IV	5
			*Observational studies:*	5

To be eligible for participation in the study, the nurses had to meet specific criteria, including being RNs, currently working as CRNs in a clinical research setting, and being actively engaged in informing patients about clinical research and conducting informed consent conversations. These criteria ensured that the participants had the qualifications, professional experience, and understanding to contribute meaningfully to the research. Six nurses declined participation due to heavy workload or recent participation in another study. Eventually, 14 CRNs (all female) participated in the study, and all held a Bachelor of Nursing and some a Master’s degree (Table 2). The participants had an average age of 43.8 years and had been RN an average of 15.8 years (with a range of one to more than 30 years) and worked 11.2 years as CRNs, ranging from less than 1 year to almost 30 years (Table 2).

**Table 2:** CRNs’ personal characteristics (*n* = 14)

Sex	*N* = 14	Age (Years)	*N* = 14	Level of education	*N* = 14	Worked as RN (years)	*N* = 14	Worked as CRN (years)	*N* = 14
Male	0								
Female	14	25–39	3	RN	8	< 1–5	5	< 1–5	4
		40–49	9	MSc Degree	6	6–10	0	6–10	3
		50–59	2			11–20	4	11–20	6
		> 60	0			21–30	3	21–30	1
						> 30	2	> 30	0

### Data collection

The semi-structured interview guide used was developed for this study. To create the most advantageous environment for the participants, they were given the flexibility to select a time and place for the interviews. These interviews could take place either in a non-clinical setting, such as the university, or digitally/over the telephone, accommodating their diverse locations across Sweden. The interview guide was pilot tested with three CRNs (included in the study), and minor adjustments were made based on feedback from these interviews. The interview questions aimed to explore the experiences and perspectives on informed consent and associated challenges (Table 3). CRNs were encouraged to share their thoughts and experiences and to give specific examples related to the recruitment process. Probing questions such as “Can you tell me more about it?” were used for deeper exploration. In this study, we embraced a comprehensive definition of “ethical challenges,” which encompasses ethical dilemmas and conflicts, while allowing CRNs to use their own definitions of what constitutes an ethical challenge. The first author, an RN and researcher with no collegial relationships with the participants, conducted the interviews between November 2022 and May 2023. The sample size was determined using the concept of information power, a pragmatic approach employed in qualitative research [24]. This approach takes into consideration various factors such as the study’s objectives, sample characteristics, theory, quality of interview dialogue, and analytical approach to guide the determination of sample sizes. The interviews lasted between 29 and 50 min, (mean 37). All interviews were recorded and transcribed verbatim. The analysis process commenced during the data collection phase [25].

**Table 3:** Interview guide

- Please introduce yourself and share some information about your role and experiences as a research nurse
- Can you describe the informed consent process in your research setting?
- What factors do you believe are important to consider when individuals are making decisions about participating in research?
- From your perspective, what are the challenges in safeguarding voluntary informed consent?
- How do you and your colleagues work to protect voluntary informed consent?
- Have you encountered any challenges in protecting voluntary informed consent in relationships where there is a dependency between the patient and the researcher?
- Based on your experiences, what suggestions or recommendations do you have for enhancing the protection of voluntary informed consent for patients involved in research?
- Is there any topic or aspect related to the informed consent process that you would like to discuss that we have not covered in this interview thus far?

### Data analysis

The qualitative data were analysed in Microsoft word and Excel using an inductive content analysis approach to describe the phenomenon in a conceptual form [26]. All authors possessed significant experience in conducting qualitative research across various research projects. The first author conducted the initial coding of the data and all authors contributed to generating the themes. The first author analysed all interviews, while the other authors independently coded and analysed three interviews each, promoting a diverse perspective. Short notes (codes) and headings were created to condense the content and highlight critical statements or concepts relevant to the study’s purpose. Following the coding process, a list of categories was organised into higher-order headings to reduce the number of categories by merging similar or dissimilar categories into broader categories, providing a description of the phenomenon. Similar categories were merged, and irrelevant categories were eliminated, resulting in a reduced total number of categories. Subcategories containing similar events and incidents were consolidated into categories, while categories were further grouped as main categories. This iterative process involved regular meetings among the research group to review and refine the categories as the analysis progressed. Discussions were held to review the analysis and reach a consensus on the categories to be utilised. The final version of the analysis was achieved through consensus and agreed upon by all authors.

## Results

The themes that derived from the data were divided into three broad categories: (1) CRNs questioning the patient’s voluntariness; (2) measures to protect research subjects; and (3) exclusion of certain groups (Table 4).

**Table 4:** Categories and subcategories identified in the analysis

Categories	Subcategories
**Threats to voluntariness**	Rushed decision-making process
	Overloaded with information
	The physician’s influence
	Disagreement within the family
	Unrealistic hope for therapeutic benefits
	Perceived insufficient knowledge to provide information
	Complexity of roles
**Measures to safeguard voluntariness**	Time to consider participation
	Advocacy and engagement
	Inclusion of vulnerable groups
**Questionable exclusion of certain groups**	Language barriers
	Impaired cognitive ability
	Worries about insufficient inclusion

### Threats to voluntariness

The first category describes various factors that pose a risk to the voluntariness of research participants. They emphasised the importance of preventing involuntariness and highlighted several factors that can contribute to participant autonomy-related vulnerability. These include factors related to the study’s design, trust, prognosis, and external influences.

#### Rushed decision-making process

CRNs repeatedly described factors that contribute to rushing patients in their decision-making process. Physicians often lack sufficient time for patients, leaving little opportunity for them to ask questions: “*the most challenging situation arises when the doctor is stressed and fails to listen to the patient*” (CRN 6). In such cases, CRNs experienced uncertainty about whether participants had been unduly influenced.

Another concern was with the protocol, which often required immediate inclusion of diagnosed patients in the study. Patients facing critical conditions or receiving new cancer diagnoses, dealing with recurring cancer or unsuccessful treatments had to make time-sensitive decisions with significant emotional weight. Consequently, CRNs observed that these circumstances hindered participants’ full comprehension of the provided information.

…the most challenging aspect is emergency studies where the patients need to make quick decisions, even when in a critical condition (CRN 12).

CRNs expressed feeling uneasy during such moments, as they believed that hurried decisions could undermine the voluntary nature of patients’ participation in research.

#### Overloaded with information

The written consent information to presumptive research subjects was perceived as excessively lengthy and detailed. The extensive content overloaded patients with information ultimately did not read or comprehend.

These consent forms and patient information, why are they so long? Is it truly necessary to list every side effect? … If they need an ultrasound, does the patient need to know that? There’s so much text, which can make you scared and the patient doesn’t have the energy to read through all of it … (CRN 3)

#### Physicians’ influence

The physicians were most often responsible for providing the initial and medical information related to the study, often without CRNs. The trusting relationship between patients and physicians played a significant role, claimed the CRNs, with patients valuing their doctors’ opinions and sometimes wanting to please them. When doctors presented a study as beneficial, highlighting its potential advantages, it greatly influenced patients’ decision-making process.

When a doctor presents a study as something beneficial, highlighting an ongoing study that could be advantageous for the patient, it can greatly influence their decision-making process. I think this can have a substantial impact on the patient’s ultimate decision regarding participation. (CRN 8)

Respondents also worried that other features of the physician‒patient relationship could interfere with the patient’s free decision-making.

Sometimes, the doctors recommend a study that they think the patients should consider since this might benefit their cancer. (Interviewer: Do you think they consent to please the doctor?) Yes, a bit like that... (CRN 11)

#### Disagreement within the family

CRNs faced ethical challenges when family members’ opinions clashed with the patient’s decision to participate in research. In some cases, relatives were insistent and eager for the patient to participate. A CRN highlighted a scenario where it appeared that relatives were primarily driving the decision for participation: “*I felt that it is mostly the relatives who pushed (for participation)*” (CRN 3). On the other hand, there were instances where relatives opposed the involvement of their sick family member in research. For example, a male patient expressed willingness to participate, but when his wife visited, she disagreed and discouraged his involvement.

Sometimes, I have conversations with male patients who express that they are willing to participate. When their wives visit, they disagree and state that he should not be participating... In such cases, I observe that she has likely influenced his decision not to participate. (CRN 12)

#### Unrealistic hope for therapeutic benefits

Most patients asked to participate in a clinical study consented, CRNs stated. Supposedly, a reason for this is that patients would not risk missing out on beneficial results, and there was a belief among the research personnel that the study would be more beneficial than ordinary treatment.

In a particular study with a new form of surgery, there can be pressure to take part, but there it is also because you believe this new method can be better. In addition, there I can imagine that... pressure would not be the right word … but you agree on possibly wrong grounds. However, what is truly right or wrong? (CRN 5)

Despite facing end-of-life situations, patients often saw participation as beneficial, clinging to hopes for therapeutic benefits or even a cure, even if their expectations were unrealistic: “*These patients, to a great extent, say yes to studies*” (CRN 8). CRNs acknowledged this ethical challenge, particularly in early intervention studies (phase I), where direct patient benefit was highly unrealistic.

Some patients have high hopes that a new drug, such as an ATMP drug [medicines for human use based on genes, tissues or cells], could provide a cure explicitly tailored for them through precision medicine. They are willing to try it, regardless of the potential costs and even if significant side effects may require ICU admission, as they believe it can offer a cure for their condition. (CRN 2)

CRNs observed that patients viewed clinical studies positively, seeing them as a valuable opportunity to improve their situation and possibly even achieve a cure, despite the odds stacked against them. CRNs involved in early-phase studies recognised the significance of supporting patients with life-threatening illnesses by participating in clinical studies. One CRN expressed that providing good care to the patient was an essential aspect of their role.

Patients might view it as an extra opportunity, an additional source of hope. Participating in studies can also benefit them, as they have additional support and assistance. However, it is important to strike a balance and not give them unrealistic expectations, but it is challenging to convey this effectively (CRN8).

#### Perceived insufficient knowledge to provide information

The informed consent process varied significantly across research centres, hospitals, studies, and study participants. Often, but not always, the physician introduced the initial information about the study to the patient; most CRNs were not part of this conversation and needed to know what information had been presented to the patient. On the other hand, CRNs were responsible for completing the entire consent process after receiving the physician’s initial information about the study.

Doctors are involved as little as possible in all studies, so to speak. They try to put as much as possible on the nurse with the follow-up visits and so on, and only at specified times the doctor comes in, or if there are any problems, of course. (CRN4)

This left CNRs alone to discuss the study with patients. However, CRNs expressed that they felt they needed more competence when trying to address all the questions from patients about the studies. This lack of confidence in their knowledge and abilities underscored the challenges faced by CRNs in fulfilling their role in the informed consent process within clinical trials.

… You get follow-up questions …How does this study drug affect and how does it interact with other drugs? Then, it can sometimes feel a little unsatisfying not to be able to answer. (CRN 9)

#### Complexity of roles

While maintaining their dedication to the research process and collaborating with physicians on informed consent, CRNs also acknowledged their critical role in participant recruitment and engagement. Nevertheless, CRNs highlighted challenging scenarios where their obligations to the study protocol or recruitment goals clashed with their primary nurse responsibilities. These dilemmas required careful navigation and reconciliation, as they were torn between their nursing responsibilities and commitment to fulfilling study requirements.

There are moments when I feel a conflict. On the one hand, we wanted to include as many patients as possible in the study. However, on the other hand, I also understand the importance of respecting their current condition and emotional state. Instead of rushing to approach them, I sometimes give them space and consideration. It is challenging to determine the right decision in such situations and what would be best for the patient. (CRN 4)

### Measures to safeguard voluntariness

The second category describes the measures taken by CRNs to protect the voluntariness of informed consent, including allowing ample time for participants to make decisions, providing continuous support and communication to address concerns and maintain engagement, and collaborating with study doctors to assess cognitive impairment and ensure informed eligibility decisions. This approach emphasised the perceived importance of prioritising participants’ well-being and inclusion in research studies and that these goals may conflict.

#### Time to consider participation

As previously mentioned, CRNs observed that most patients conceded to inclusion in studies. Since CRNs sometimes found the inclusion process too rapid, they sought to safeguard time for it. Therefore, the CRNs needed to let patients have enough time to consider participation.

It is crucial to avoid making hasty decisions, allowing sufficient time for the research participant or patient to read the consent document thoroughly. Creating a peaceful and quiet environment encourages thoughtful consideration of their participation. This step is of utmost importance, as it sets the foundation for approaching the clinical trial with the right attitude. Additionally, being well informed and educated about the expectations and potential side effects reduces the risk of dropout. Therefore, thorough preparation is pivotal in ensuring the research participant’s commitment to remain in the study. (CRN 7)

CRNs underscored the importance of maintaining a patient-centred approach in research and their role in prioritising the well-being of patients while also emphasising the significance of stepping out of their comfort zones when faced with challenges that may impact patient recruitment and the integrity of the research process. These situations mainly arose when research colleagues stressed the importance of enrolling more participants, while the CRNs played a crucial role in advocating for the patients’ well-being and ensuring adequate time for deciding whether to participate.

I have noticed a trap that some colleagues fall into, where they do not fully recognise that these studies are not ours. As research nurses, our responsibility lies with the patients and ensuring their proper inclusion in studies. While the number of patients included can be part of our job description, it should not be a source of stress or lead to hasty and inadequate inclusions due to unrealistic expectations. In such cases, being assertive and willing to step out of our comfort zone becomes necessary. (CRN 5)

CRNs described the importance of being highly knowledgeable about the research protocol, as it serves as their primary tool for work. They regularly reviewed the ethics application and adhered to it diligently, especially when confronted with questions that may require deviating from the approved procedures.

What I see is... it’s written in the protocol for the ethics application how recruitment should be conducted, and then you start to deviate from it… The physician asked me, “we have not heard anything from this patient, can you call or send out a reminder letter?” I cannot do that because we do not have ethics approval. It is stated that when you meet the patient at the reception or the ward, you shall inform them and then ask. If the patient is unsure about the study or has not explicitly said no, then the physician can ask me to deviate from the approved protocol and send a reminder or make a call. (CRN 6)

#### Advocacy and engagement

CRNs acknowledged the potential value of performing research to the benefit of patients. They provided extended support and resources to research study participants, recognising the importance of maintaining their engagement and addressing any concerns that may arise. Furthermore, they found themselves in a critical role as advocates for patients. They emphasised the significance of understanding patients’ perspectives and reasons for potentially withdrawing from the study, aiming to delve deeper into the underlying issues and find suitable resolutions. CRNs highlighted the significance of ongoing communication and follow-up, even after the study’s completion, to ensure the participants’ well-being and gather complete data for the study’s validity and outcomes.

We try to keep patients in the study, of course... and that is where we research nurses become very important. We might ask the reason for wanting to quit to understand their perspective and what quitting means to them. It allows us to address the underlying issues and truly understand their concerns. Sometimes, patients initially say no, but they want to participate. It could be that they were overwhelmed by the information received and they found it easier to say no at that moment. When talking about it and delving deeper into the matter, they might realise that continuing is easier than they initially thought. (CRN 12)

Additionally, they offered additional support and guidance to address any concerns or difficulties faced by participants. Patients rarely expressed a desire to withdraw, but when doing so, they put CRNs in the difficult position of balancing adherence to the study protocol with respect to participants’ choices.

#### Inclusion of vulnerable groups

CRNs described a collaborative approach in addressing uncertainties regarding participants’ cognitive impairment, seeking guidance from the PI to determine their eligibility for enrolment. They emphasised the importance of thorough evaluations conducted by the study doctor to make informed decisions, highlighting the significance of collaborative decision-making processes in research studies.

Suppose you suspect it could be early signs of dementia or another condition. In that case, we always consult with the study doctor for a thorough evaluation, and the doctor performs the final assessment (CRN13).

### Questionable exclusion of certain groups

The third category explores the recognition by CRNs of the vital importance of equitable participation in clinical studies. They acknowledged that language barriers, cognitive limitations, and disabilities often posed notable difficulties during the informed consent process. These groups were often excluded from research studies, and the CRNs perceived it as an ethical dilemma, as many individuals within these groups may value participation. They believed that this hindered essential research from being conducted within these populations.

#### Language barriers

When asked, the CRN described that language barriers could be a significant obstacle to participation, with not speaking Swedish being a common exclusion criterion: “*It is problematic that we exclude those who cannot speak or write Swedish because they are still within our context…*” (CRN 4). One CRN expressed concerns about the potential consequences of excluding certain groups, emphasising that it could lead to a lack of diversity and inadequate representation of those populations in research.

It is funny that you bring this up because we have had several recent discussions, particularly regarding specific patient groups where certain diseases are more prevalent among foreign-born individuals… One aspect that needs to be considered is the staff’s ability to communicate in languages other than their own effectively. (CRN 7)

#### Impaired cognitive ability

CRNs mentioned those with impaired cognitive abilities as another underrepresented group in research studies. CRNs acknowledged that these factors often posed significant obstacles in the informed consent process and expressed concern that many individuals within these vulnerable groups were not even being approached to participate.

In most studies, it (impaired cognitive ability) is listed as an exclusion criterion. However, if it is not specified for some reason, then we take it into consideration … However, if they are unable to continue, they would have to be withdrawn from the study, of course. (CRN 10)

When there was uncertainty regarding a person’s cognitive ability, CRNs would request the PI to assess their capacity and determine if they could be appropriately informed about the study. However, CRNs expressed concerns about the need for more available resources for assessing decision-making capacity or providing sufficient support in that regard.

#### Worries about insufficient inclusion

CRNs expressed concern regarding researchers’ potential hesitancy to approach these individuals, which could lead to limited access to innovative treatments and exclusion from clinical trials, potentially resulting in premature deaths. They also highlighted concerns about the potential implications for the generalizability of research findings and the equitable distribution of benefits.

I believe that certain groups, such as those with, e.g. autism, Asperger’s, ADHD, or … cognitive disabilities, are underrepresented. They (researchers) may hesitate to approach these individuals for participation, resulting in limited access to cutting-edge cancer drugs and exclusion from certain trials … these groups die prematurely as they are not allowed to participate in studies. (CRN 2)

## Discussion

This study focused on CRN challenges in the informed consent process in clinical research. Based on the findings, this discussion elucidates important areas concerning ethical challenges in clinical research.

When participating in the informed consent process, CRNs saw rushed decision-making, low comprehension of complex consent forms, and too much trust in the physician’s recommendations. Despite these challenges, CRNs reported that the majority of patients expressed willingness to participate in clinical research, with only a small number choosing to withdraw. This aligns with the high motivation and willingness to participate in research reported previously, particularly in oncology trials [27, 28]. However, barriers to participation, such as fear and distrust, have been observed, especially among minority ethnic groups [29]. In this Swedish study, general trust is high in the healthcare system, and the fact that nonfluent Swedish speakers were regularly excluded may have contributed to the observed high inclusion rates.

The literature notes that rushed decision-making, low comprehension of information and trust risk being detrimental to participant understanding, thus undermining voluntariness even though motivation to participate is high. A systematic review reported that while many research subjects rate their subjective knowledge as high, objective evaluations show that few genuinely deeply understand the research [11]. Many participants admit to not fully reading the information and feeling hesitant to ask questions, which raises concerns about their authentic involvement in shared medical decision-making, as noted by Pietrzykowski et al. [11].

In this study, CRNs observed that information provided by physicians, whether presented optimistically or not, was not neutrally received. Patient interests heavily influenced their understanding of the information. While challenges may be less prominent in certain low-risk and nonsensitive clinical research areas, they can become more pronounced in early-phase research or when patients face vulnerability due to life-threatening conditions. To counteract ethical fading in end-of-life care, where hope for therapeutic benefits and trust in physicians can overshadow ethical considerations, CRNs need to be aware of the vulnerability and desperation experienced by patients with life-threatening diseases [30].

In our study, CRNs implemented protective measures, such as providing sufficient decision-making time and support, particularly for terminally ill patients. There are more measures needed, however. A systematic review by Hillersdal et al. revealed that family influence, therapeutic hopes, and existential needs were often overlooked when supporting patient decision-making in oncology trials [31]. They emphasise the importance of interventions considering person-centred factors beyond improving trial knowledge. Therefore, CRNs should also incorporate patient preferences and the social context of decision-making processes in their practical and moral deliberation processes.

In our interviews, we found that CRNs often hesitate to accept the usual corollary of a right to abstain from participation for any reason: to not ask why a patient says no to further participation in a study. This seems acceptable from a relational perspective where we make our decisions with concern for others of importance to us. Therefore, a dialogue based on respect, transparency, and collaboration becomes essential to ensure that participants actively engage in the decision-making process for the sake of research, themselves and significant others. This approach recognises the importance of CRNs fostering a dialogue that values their input and addresses their concerns, where researchers can uphold ethical principles and maintain the integrity of the research process [32].

The CRNs in our study faced a tension between their obligations as nurses and the scientific requirements of conducting clinical research, echoing findings from other studies. Larkin et al. found that CRNs faced ethical challenges due to dual obligations and that such conflicting loyalties can lead to both internal (e.g. own belief system) and external conflicts (e.g. lack of time, complex consent form) [18]. CRNs in this study had to handle conflicts between the demands of the protocol and the inherent goal of maintaining the nurse‒patient relationship. Balancing adherence to the protocol with personalised patient care is crucial for conducting ethical and effective research [33]. Straying too far from the established protocol to meet individual patient needs may potentially jeopardise the study’s reliability and accuracy, while adhering strictly to the protocol without considering personalised care can harm participants’ well-being. To address this challenge, CRNs need to strike a balance that permits flexibility while upholding the study’s core principles [34]. CRNs can ensure that participant needs are met without compromising scientific rigour by carefully considering and documenting any deviations from the protocol and their justifications. The CRNs in this study expressed that they tried to tailor to the person’s needs and context when participants wanted to withdraw from the study. The CRNs had in mind the right to withdraw without any negative consequences or pressure to continue participation. This approach enabled CRNs to demonstrate empathy and responsiveness to each participant’s unique needs while contributing to scientific advancement. Ultimately, successfully striking this balance ensures that research studies uphold both scientific standards and the well-being of participants. Conversely, if ethical challenges are left unresolved, it can cause *moral distress* and detachment among CRNs, leading to negative consequences [35].

Another finding in this study is that CRNs perceive that individuals with language barriers or cognitive disabilities often face exclusion from research. The absence of diversity in clinical trials presents moral, scientific, and medical concerns that this unnecessarily limits people’s access to potentially life-saving treatments and makes them unable to realise altruism in the form of research participation [36]. This limited representation of diverse groups in clinical research also hinders scientific knowledge advancement by reducing the external validity of medical research [37]. One would think the information materials to more languages could be a solution. However, it is costly, and translating research materials has been shown to present its own challenges, requiring accurate and effective communication across linguistic and cultural boundaries. Brelsford et al. found problems such as non-equivalent registers, errors of omission, and changes that altered substantive meaning in translated consent materials [38]. To remedy the situation, we suggest that working with translators with linguistic expertise and understanding sociocultural factors is crucial.

Similarly, individuals with disabilities are often excluded from clinical research, and the criteria for exclusion lack justification [39]. A study by DeCormier et al. examined protocols on ClinicalTrials.gov and discovered that while most studies allowed broad eligibility, few explicitly permitted individuals with cognitive disabilities to participate with support [39]. Across various domains, eligibility criteria frequently exclude individuals with disabilities, including those with psychiatric or cognitive impairments. The study authors recommend closely examining eligibility criteria, providing scientific or ethical justifications, and implementing accessible study designs. To address these issues, we believe it to be important also that CRNs should receive support and aids to facilitate interactions with these groups. Thereby, ensuring inclusivity and diversity in clinical research, CRNs can play a pivotal role in actively involving a more comprehensive range of underrepresented populations. This involves meaningful engagement with patients, the public, and communities while safeguarding the rights and well-being of all participants [40].

### Strengths and limitations

To enhance the trustworthiness of this qualitative study, the authors implemented several strategies to improve credibility, dependability, and transferability [41]. Credibility was established by utilising multiple interviews from many different research contexts and providing a comprehensive description of the research context and participants. Regular meetings among the authors were held to reach a consensus and maintain transparency throughout the analysis process, reducing bias and strengthening confirmability. Dependability was strengthened by employing an interview guide and ensuring that one author conducted all the interviews, ensuring consistency in data collection. To enhance transferability, the study included research nurses with different years of experience as CRNs from various medical fields and diverse clinical units, spanning both pharmaceutical and academic studies, interventions and observational research, various trial phases, and a wide range of patient populations and diseases in Sweden, which provided a broader representation of perspectives. Furthermore, to assist readers in assessing the applicability of the findings to their context, the authors aimed for transparency in all study steps. This study also carries several limitations worth noting. First, it is essential to acknowledge that all participating CRNs were women, which might be a limitation. Second, the experiences of CRNs are derived from a relatively small sample (*N* = 14). Expanding the sample size and including males could potentially offer additional insights into the ethical challenges encountered by CRNs.

## Clinical implications

- CRNs should be mindful of time constraints and overwhelming information during the informed consent process, ensuring that patients understand comprehensively before making decisions.
- CRNs need to balance respecting patient autonomy and adhering to scientific requirements, ensuring personalised patient care while upholding the study’s core principles.
- Efforts should be made to encourage the participation of underrepresented groups in research, particularly among minority ethnic groups and those with cognitive disabilities, to enhance the possibility that patients will participate in clinical research.

## Conclusions

In conclusion, upholding ethical research standards requires recognising various factors affecting patient voluntariness. Researchers and CRNs should prioritise refining the informed consent process, overcoming participation challenges, and aligning scientific rigour with personalised care. Additionally, a concerted effort is vital to meet the diverse needs of patient populations, including equitable inclusion of individuals with language barriers or cognitive limitations in clinical studies. These findings have significant implications for enhancing clinical research integrity and advancing person-centred care.

### Supplementary Information

**Additional file 1: Research checklist.** Consolidated criteria forreporting qualitative studies (COREQ): 32-item checklist.

## Data Availability

The datasets used and analysed during the current study are available from the corresponding author upon reasonable request.

## Abbreviations

CI: Clinical investigator
CRN: Clinical research nurse
PI: Principal investigator
RCT: Randomised clinical trial
RN: Registered nurse
