# Supplementary material for: Challenges regarding informed consent in recruitment to clinical research: a qualitative study of clinical research nurses’ experiences
Source: Trials. 2023 Dec 11;24:801. doi: 10.1186/s13063-023-07844-6 (PMC10712041; doi:10.1186/s13063-023-07844-6)
Supplement: Supplementary file 1 — Additional file 1: Research checklist. Consolidated criteria forreporting qualitative studies (COREQ): 32-item checklist. [file 13063_2023_7844_MOESM1_ESM.docx]

**Research checklist**

Consolidated criteria for reporting qualitative studies (COREQ): 32-item checklist

| **Domain 1: Research team and reflexivity** | | |
| --- | --- | --- |
| Personal Characteristics |  |  |
| 1. Interviewer | TG | Page 8 |
| 2. Credentials.  What were the researcher's credentials? | TG: RN, PhD  NJ: PhD  JB: MD, PhD |  |
| 3. Occupation.  What was their occupation at the time of the study? | TG: Associate professor, Researcher  NJ: Professor in clinical medical ethics  JB: MD in palliative care, Researcher |  |
| 4. Gender.  Was the researcher male or female? | TG: Female  NJ: Male  JB: Male |  |
| 5. Experience and training.  What experience or training did the researcher have? | TG: considerable experience with qualitative research, for a variety of research projects, one of them concerned her PhD thesis (interviews with terminally ill patients in phase I trials).  NJ: considerable experience with qualitative research and leading discussion groups (moral deliberation), member of different ethical committees.  JB: experience with qualitative research, one of them concerned his PhD thesis and experiences of leading ethics discussion groups.  All authors possessed significant experience in conducting qualitative research across various research projects. | Page 9 |
| Relationship with participants |  |  |
| 6. Relationship established  Was a relationship established prior to study commencement? | The researcher had no collegial relationships with the participants. The relationship was established with a few CRNs before study commencement. | Page 8 |
| 7. Participant knowledge of the interviewer  What did the participants know about the researcher? | Some knew that TG had done interviews with patients that participated in clinical trials (PhD). | Page 8 |
| 8. Interviewer characteristics  What characteristics were reported about the interviewer/facilitator? | That TG is interested in the subject of informed consent, and this interest is shared by the entire research group. |  |
| **Domain 2: study design** | | |
| Theoretical framework |  |  |
| 9. Methodological orientation and Theory  What methodological orientation was stated to underpin the study? | Inductive content analysis. | Page 9 |
| Participant selection |  |  |
| 10. Sampling  How were participants selected? | Purposive snowball sampling. | Page 6 |
| 11. Method of approach  How were participants approached? | email | Page 6 |
| 12. Sample size  How many participants were in the study? | 14 | Page 7 |
| 13. Non-participation  How many people refused to participate or dropped out? Reasons? | Six nurses declined participation. No dropout. The reason concerned heavy workload and recent participation in another study. | Page 7 |
| 14. Setting of data collection  Where was the data collected? | They were given the flexibility to select a time and place for the interviews. These interviews could take place either in a non-clinical setting, such as the university, or digitally/over the telephone, accommodating their diverse locations across Sweden. | Page 8 |
| 15. Presence of non-participants  Was anyone else present besides the participants and researchers? | To the interviewer’s knowledge no one else were present besides the participants and researchers. |  |
| 16. Description of sample  What are the important characteristics of the sample? | Large variety. See tables on respondent characteristics in main document. | Page 7-8 |
| Data collection |  |  |
| 17. Interview guide Were questions, prompts, guides provided by the authors? Was it pilot tested? | Yes, semi structured. Yes, it was pilot tested.  Probing questions such as "Can you tell me more about it?" were used. | Page 8 |
| 18. Repeat interviews. Were repeat interviews carried out? If yes, how many? | No. |  |
| 19. Audio/visual recording.  Did the research use audio or visual recording to collect the data? | Yes, we used an audio recorder. | Page 8 |
| 20. Field notes  Were field notes made during and/or after the interview or focus group? | No additional field notes were taken. |  |
| 21. Duration.  What was the duration of the interviews or focus group? | The interviews lasted between 29 and 50 minutes (mean 37). | Page 8 |
| 22. Data saturation.  Was data saturation discussed? | We used the concept of information power, a pragmatic approach employed in qualitative research. | Page 8 |
| 23. Transcripts returned. Were transcripts returned to participants for comment and/or correction? | No. |  |
| **Domain 3: analysis and findings** | | |
| Data analysis |  |  |
| 24. Number of data coders.  How many data coders coded the data? | The first author conducted the initial coding of the data and, while the other authors independently coded and analysed three interviews each. | Page 9 |
| 25. Description of the coding tree. Did authors provide a description of the coding tree? | Table 4 displays the categories and subcategories identified in the analysis codes. | Page 10 |
| 26. Derivation of themes.  Were themes identified in advance or derived from the data? | Derived from the data. | Page 11 |
| 27. Software.  What software, if applicable, was used to manage the data? | Microsoft word and Excel. | Page 9 |
| 28. Participant checking.  Did participants provide feedback on the findings? | No |  |
| Reporting |  |  |
| 29. Quotations presented.  Were participant quotations presented to illustrate the themes / findings? Was each quotation identified? | Yes. All identifiers were removed from the transcribed interviews and pseudonymized with a code to ensure that identification of interviewees was not possible. | Page 28 |
| 30. Data and findings consistent.  Was there consistency between the data presented and the findings? | The authors have made an effort to keep the findings close to the original data and the interviewer recognized the discussions in the final results presentation. | Page 10 |
| Were major themes clearly presented in the findings? | The major categories are described in the Results as well as in Table 4. | Page 10 |
| 32. Clarity of minor themes.  Is there a description of diverse cases or discussion of minor themes? | The minor themes are described in the text as well as in Table 4. | Page 10 |
